# Case Report: Acute Heart Failure Induced by the Combination of Takayasu's, Takotsubo and Coronary Vasospasm in an Elementary School Teacher–A Reaction to Return-to-Work Stress After COVID-19?

**DOI:** 10.3389/fpsyt.2022.882870

**Published:** 2022-05-02

**Authors:** Inês Pires, Massimo Mapelli, Nicola Amelotti, Elisabetta Salvioni, Cristina Ferrari, Andrea Baggiano, Edoardo Conte, Irene Mattavelli, Piergiuseppe Agostoni

**Affiliations:** ^1^Cardiology Department, Centro Hospitalar Tondela-Viseu, Viseu, Portugal; ^2^Centro Cardiologico Monzino, IRCCs, Milan, Italy; ^3^Cardiovascular Section, Department of Clinical Sciences and Community Health, University of Milan, Milan, Italy

**Keywords:** Takayasu's arteritis, Takotsubo syndrome, coronary vasospasm, myocardial infarction with non-obstructive coronary arteries (MINOCA), emotional stress, catecholamines, case report

## Abstract

**Introduction:**

Takayasu's arteritis (TA) is a systemic inflammatory disease that affects aorta and its major branches. There are several cardiac manifestations of TA and an association with Takotsubo syndrome (TTS) – but not coronary vasospasm - has been previously reported. The role of emotional stress in this context is unknown.

**Case presentation:**

A 58-year-old Caucasian female elementary school teacher, with a history of generalized anxiety disorder (GAD), severe asymptomatic aortic regurgitation (AR), and TA in remission under corticosteroids, was admitted in the emergency department with worsening chest pain and dyspnea, initiated after a period of intense emotional stress (increased workload during COVID-19 pandemic). Physical examination revealed signs of heart failure (HF) with hemodynamic stability and an early diastolic heart murmur. The electrocardiogram showed sinus tachycardia, T wave inversion in left precordial and lateral leads, and a corrected QT of 487 ms. Laboratorial evaluation presented high values of high-sensitivity troponin I (3494 ng/L) and B-type natriuretic peptide (4759 pg/mL). The transthoracic echocardiogram revealed severe dilation of left ventricle (LV) with moderate systolic dysfunction, due to apical and midventricular akinesia, and severe AR. The coronary angiography showed normal coronary arteries. An acetylcholine provocative test induced spasm of both the left anterior descending and circumflex arteries, accompanied by chest pain and ST depression, completely reverted after intracoronary nitrates administration. The patient was switched to diltiazem and a drug multitherapy for HF was started. A cardiac magnetic resonance revealed severe dilation of the LV, mild apical hypokinesia, improvement of ejection fraction to 53%, signs of myocardial edema and increased extracellular volume in apical and mid-ventricular anterior and anterolateral walls, and absence of myocardial late gadolinium enhancement, compatible with TTS. At discharge, the patient was clinically stable, without signs of HF, and a progressive reduction of troponin and BNP levels was observed. A final diagnosis of TTS and coronary vasospasm in a patient with GAD and TA was done.

**Discussion:**

We present the first case of acute HF showing coexistence of TA, TTS and coronary vasospasm. TA is a rare inflammatory disease that can be associated with TTS and coronary vasospasm. Besides that, coronary vasospasm may also be involved in TTS pathophysiology, suggesting a complex interplay between these diseases. Mood disorders and anxiety influence the response to stress, through a gain of the hypothalamic-pituitary-adrenal axis and an increased cardiovascular system sensitivity to catecholamines. Therefore, although the mechanisms behind these three pathologies are not yet fully studied, this case supports the role of inflammatory and psychiatric diseases in TTS and coronary vasospasm.

## Introduction

Takayasu's arteritis (TA) is a systemic inflammatory condition that affects medium and large arteries, mainly aorta and its major branches, and leads to stenosis, occlusions or aneurysmal degeneration of these vessels (1). There are several cardiac manifestations of TA and recently a relationship between this pathology and Takotsubo syndrome (TTS) has been described (2).

TTS is an acute cardiac syndrome characterized by typical wall motion abnormalities leading to a usually transient systolic dysfunction in the absence of culprit epicardial coronary artery disease and is triggered by emotional or physical stressors. The exact pathophysiology of TTS is unknown, but there is evidence that intense sympathetic activation and high catecholamine levels play a role, leading to myocardial stunning possibly through coronary artery spasm and microvascular dysfunction, among other factors (3).

We present a case of a female patient with history of TA and generalized anxiety disorder (GAD), that, in response to an intense emotional stress, developed a myocardial infarction with non-obstructive coronary arteries (MINOCA) with signs of heart failure (HF). In addition to electrocardiogram and transthoracic echocardiogram changes suggestive of TTS, we documented a multivessel coronary vasospasm during an invasive provocative test.

## Case Description

A 58-year-old Caucasian female was admitted in the emergency department complaining of sudden onset dyspnea and retrosternal chest pain, radiating to the left arm, that appeared at rest and lasted for several h. The patient was a teacher and reported a recent period of intense work-related emotional stress, after having returned to presential classes during the COVID-19 pandemic and having extra work to replace quarantined colleagues. She had history of GAD, TA in remission under treatment with low-dose corticosteroids, severe asymptomatic aortic regurgitation (AR) with left ventricular (LV) dilation and ejection fraction (EF) of 56%. Moreover, in the past she suffered from crisis of angina with previous documentation of a left anterior descending (LAD) artery myocardial bridging; paroxysmal supraventricular tachycardia; uterine myomas with recurrent bleeding and necessity of uterine artery embolization. In addition to prednisone, her chronic medication included bisoprolol, flecainide, transdermic nitrates and vitamin supplements. There was no relevant family history.

**Figure 1:**
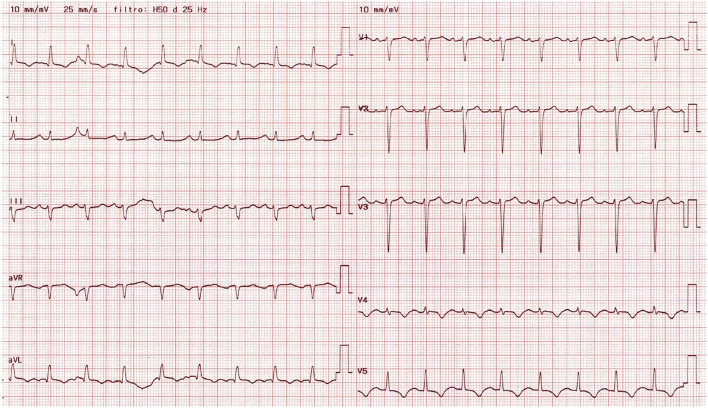
12-lead electrocardiogram obtained at admission, showing sinus tachycardia, T wave inversion in left precordial, inferior and lateral leads, and a prolonged corrected QT.

**Figure 2:**
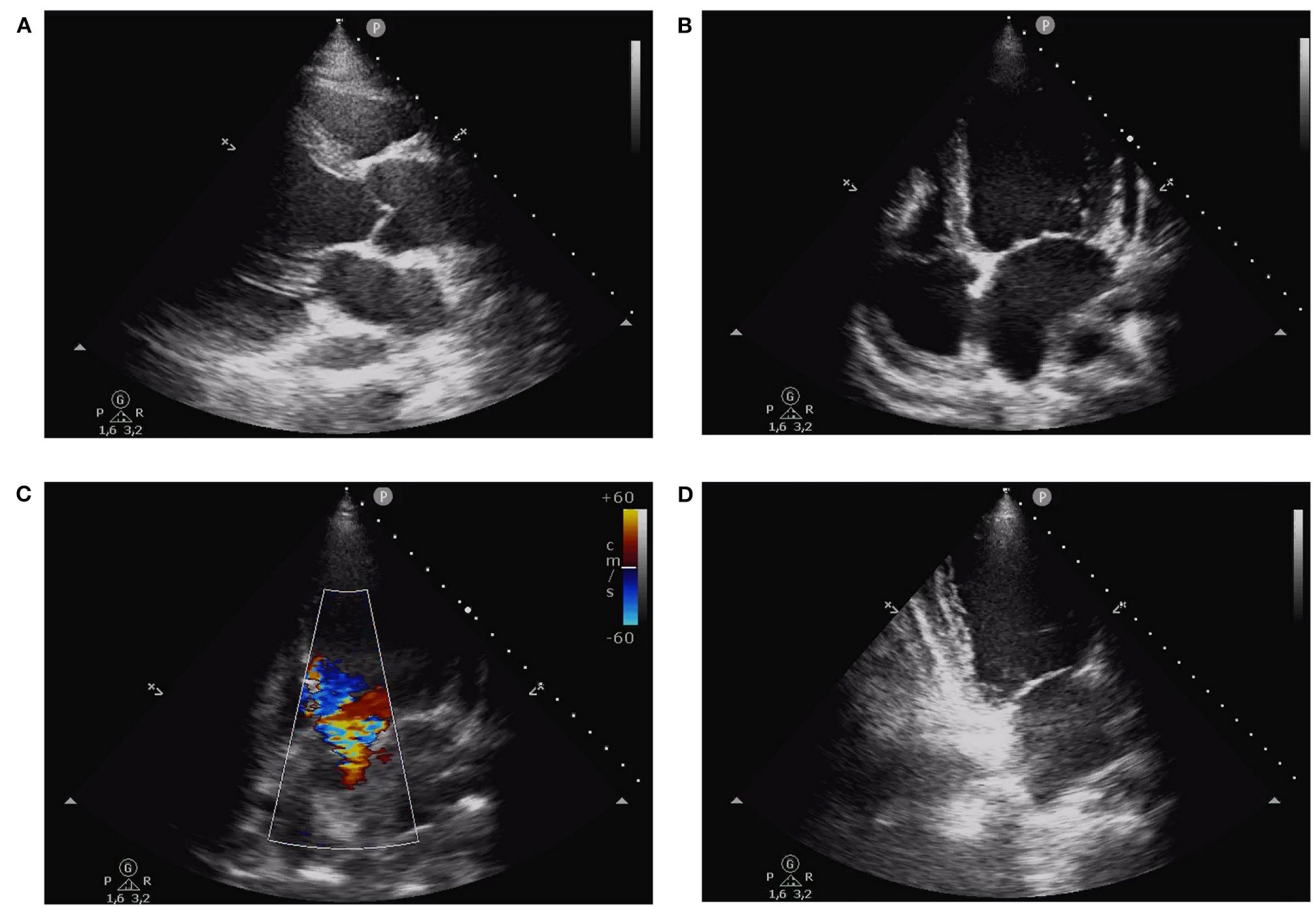
Transthoracic echocardiogram obtained at admission. **(A)** Parasternal long axis view showing left ventricle dilation and dilation of aortic root and ascending aorta. **(B)** Apical four-chamber view revealing left ventricle dilation with reduced ejection fraction due to apical and midventricular akinesia in the septal and lateral walls, and normal right ventricular function. **(C)** Severe aortic regurgitation in (five-chamber view). **(D)** Apical two-chamber view showing akinesia of the midventricular and apical segments of the anterior and inferior left ventricular walls.

Physical examination revealed blood pressure of 120/40 mmHg, tachycardia (110 bpm), symmetrical and bounding peripheral pulses with no blood pressure differential and oxygen saturation of 98% on room air. The patient was eupneic, had mild basal crackles at pulmonary auscultation and an early diastolic heart murmur on cardiac auscultation. There were no signs of peripheral hypoperfusion. The electrocardiogram revealed sinus tachycardia, T wave inversion in left precordial leads, inferior and lateral leads, and a corrected QT of 487 ms (Figure 1). The chest X-ray was unremarkable. Laboratorial evaluation showed hemoglobin of 11.3 g/dL, high sensitivity troponin I of 3494 ng/L and B-type natriuretic peptide of 4759 pg/mL, with no other alterations. The transthoracic echocardiogram revealed severe LV dilation with reduced EF (36%), due to apical and midventricular akinesia; severe AR; dilation of aortic root and ascending aorta (23 mm/m2 and 24 mm/m2 respectively); moderate tricuspid regurgitation; maximal tricuspid regurgitation velocity of 3.3 m/s; and right ventricle with normal dimensions and longitudinal systolic function (Figure 2/Supplementary Video 1).

**Figure 3:**
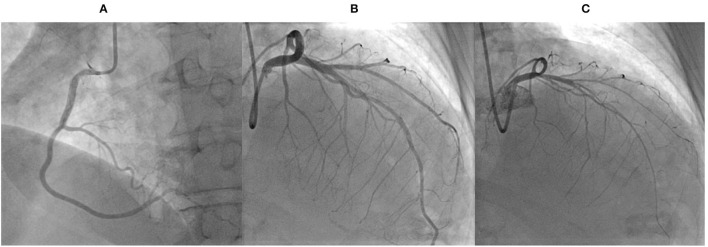
Coronary angiography and acetylcholine test performed during admission. **(A)** Coronary angiography in the left anterior oblique view showing normal right coronary artery. **(B)** Cranial right anterior oblique view showing normal left coronary artery. **(C)** Cranial right anterior oblique view revealing spasm of both the left anterior descending and circumflex arteries, during an acetylcholine provocative test.

The patient was admitted to the Cardiac Intensive Care Unit (ICU) with a suspicion of non-ST elevation myocardial infarction. A cardiac computed tomography (CCT) showed a previously known myocardial bridging in the distal LAD, with an extent of 19 mm and a depth <2 mm, without coronary artery disease. The diagnosis of MINOCA was made. During ICU stay, the patient had recurrent transient chest pain, with worsening of ST segment depression in lateral leads. Due to recurrence of symptoms, it was decided to perform a coronary angiography that confirmed the absence of obstructive coronary arteries disease. During cannulation of the left coronary artery, an acetylcholine provocative test was performed, showing spasm of both the LAD and circumflex arteries, accompanied by reproduction of chest pain and ST depression, which completely reverted after intracoronary nitrates administration (Figure 3). The patient was switched to diltiazem, while nitrates and beta-blockers were stopped. Afterwards, there was no chest pain recurrence, and the patient maintained hemodynamic stability with no ventricular arrythmias. Due to the moderate systolic dysfunction and high BNP levels, levosimendan was administered, and disease modifying drugs for HF, namely angiotensin receptor-neprilysin inhibitor (ARNI) and mineralocorticoid receptor antagonists were started 2 days after admission. There was good tolerance to sacubitril/valsartan and spironolactone, without side effects, worsening of renal function or hyperkalemia. Due to anxiety, the patient received continuous psychological support and low-dose anxiolytic drugs (sertraline 50 mg id and alprazolam 0.5 mg id) during her hospital stay.

A cardiac magnetic resonance performed on the 9^th^ day after admission revealed severe dilation of the LV, mild apical hypokinesia, improvement of EF to 53%, signs of myocardial edema and increased extracellular volume in apical and mid-ventricular anterior and anterolateral walls, and absence of myocardial late gadolinium enhancement, compatible with TTS. This exam also showed severe aortic regurgitation, aortic dilation, and increased parietal thickness, slight edema and late gadolinium enhancement in the ascending aorta, consistent with TA.

At discharge, the patient was clinically stable, with no signs of HF, with progressive reduction of troponin and BNP levels and with electrocardiogram showing persistent T wave inversion and stable QTc. Disease-modifying drugs for HF, namely sacubitril/valsartan 24/26 mg bid, were maintained at discharge as they probably contributed to the improvement in EF and to the reverse remodeling. After a discussion with the patient, it was decided to wait for the surgical aortic valve replacement, and she was referred to psychiatry and cardiology short-term follow-up. A final diagnosis of MINOCA (with transient acute HF) due to coronary vasospasm and TTS, induced by emotional stress, in a patient with TA and GAD was done. Patient's timeline is shown in Table 1.

**Table 1:** **Timeline**.

Background	Takayasu's arteritis Severe aortic regurgitation Generalized anxiety disorder
Seven days before admission	Period of intense work-related emotional stress, after returning to work during the COVID-19 pandemic
Day zero (admission)	Chest pain and dyspnea. Elevation of troponin and B-type natriuretic peptide. Transthoracic echocardiogram revealing severe left ventricle dilation with reduced ejection fraction, due to apical and midventricular akinesia.
Day one	Cardiac computed tomography showing normal coronary arteries.
Day two and 3	Recurrence of chest pain. Coronary angiography with absence of obstructive coronary arteries disease. Acetylcholine provocative test showing spasm of the left coronary artery, accompanied by reproduction of chest pain and ST depression.
Day nine	Cardiac magnetic resonance revealing severe dilation of the left ventricle, mild apical hypokinesia, improvement of ejection fraction to 53%, signs of myocardial edema and increased extracellular volume in apical and mid-ventricular anterior and anterolateral walls, and absence of myocardial late gadolinium enhancement, compatible with Takotsubo syndrome.
Day 10	Discharge after optimization of disease modifying drugs for heart failure, namely angiotensin receptor-neprilysin inhibitor (ARNI) and mineralocorticoid receptor antagonists. The patient also received continuous psychological support and low-dose anxiolytic drugs during hospital stay.

## Discussion

**Figure 4:**
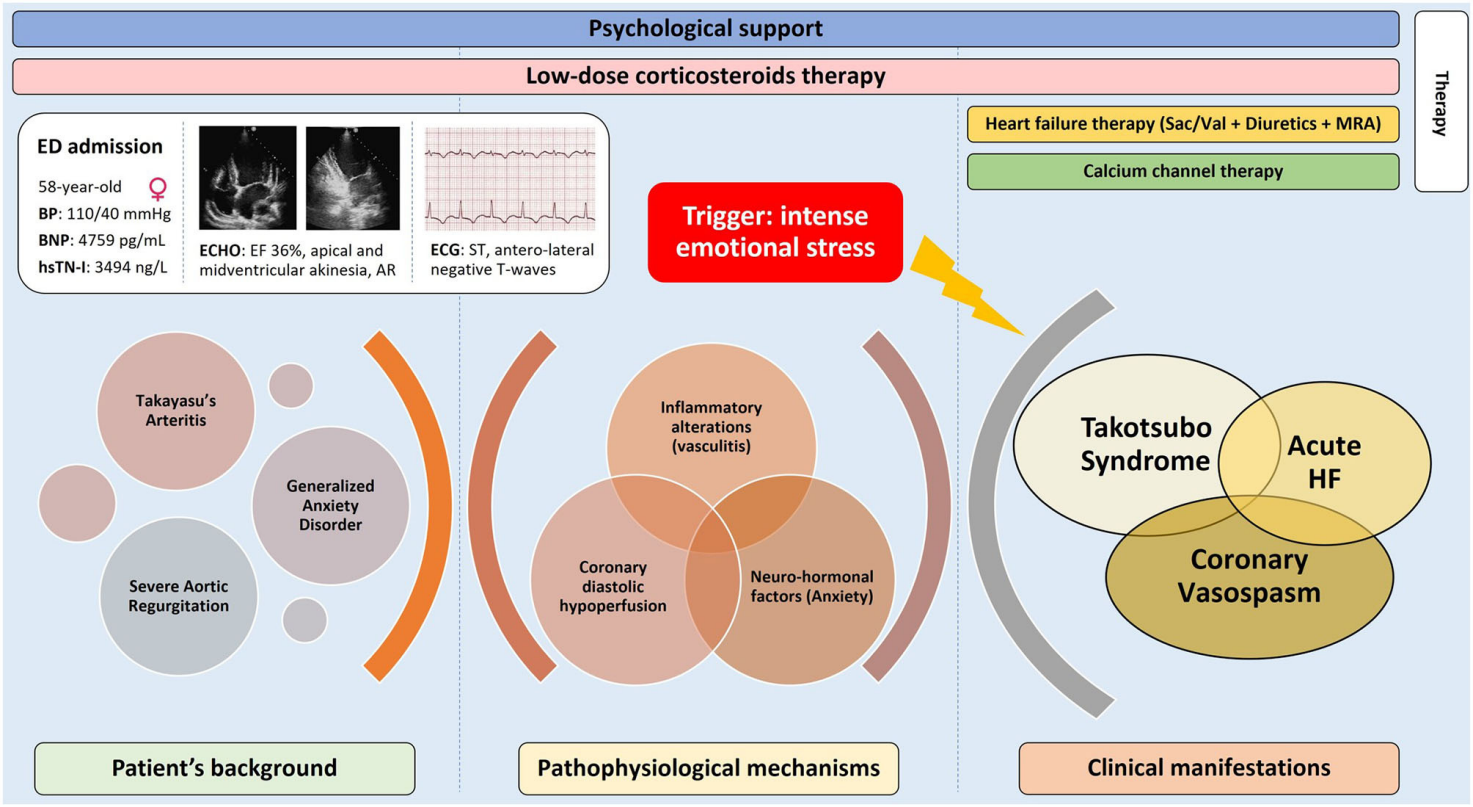
Central illustration showing the complex interplay between patient background conditions (Takayasu's, aortic regurgitation and generalized anxiety disorder), underlying pathophysiological mechanisms and clinical manifestations, including acute heart failure, Takostubo syndrome and coronary vasospasm, triggered by an intense emotional stress. The clinical presentation, main complementary exams and the therapy during admission are outlined.

We presented a case of MINOCA in a 58-year-old Caucasian woman in which the complex interplay between different conditions (GAD, autoimmune diathesis, aortic valve disease, coronary vasospasm) resulted in an acute HF syndrome with myocardial disfunction, left ventricle ischemia and troponin release (Figure 4). Although speculative, this clinical picture suggests a cumulative role of different pathophysiological pathways initially triggered by an intense emotional stress.

TA, our patient's main background diagnosis, is a chronic vasculitis involving large vessels, that predominantly involves the aorta and its branches. Although its cause is unclear, there is a strong association with genetic predisposition, environmental factors, and the role of microbes. Pathogenic activated T-lymphocytes and macrophages, as well as proinflammatory cytokines, lead to granulomatous inflammation and vessel wall damage. TA predominantly affects women in the second and third decades of life and, although it is more prevalent in Asians, it has a worldwide distribution (1).

Cardiac involvement in TA can reach 40% of patients and can affect virtually any structure of the heart, with an associated worsening in prognosis. Cardiac manifestations include valvular abnormalities (mainly AR), myocardial abnormalities including HF or myocarditis, coronary artery disease, pericardial effusion or pericarditis, among other rarer presentations (4). The frequency of coronary artery involvement in TA is 10–30% and the three most common lesions are stenosis or occlusion of the coronary ostia (60–80%), diffuse disease that may involve all epicardial branches or only focal segments (10–20%) and coronary aneurysms (0–5%) (5, 6). However, TA patients can also present with typical angina with normal coronary arteries, secondary to diastolic hypotension and reduced coronary blood flow (7). In this regard, diastolic hypoperfusion due to the presence of concomitant severe AR, as observed in our case, could be an additional mechanism leading to myocardial ischemia.

Moreover, Lim et al. recently reported the first case of a patient presenting with TTS in which TA was diagnosed (2). TTS is an acute cardiac syndrome characterized by typical wall motion abnormalities leading to transient LV systolic dysfunction in the absence of culprit epicardial coronary artery disease (3). Although there are several anatomical TTS variants, the most common type is the apical ballooning type, also known as the “typical” form, in which the LV apical segments present hypokinesia, akinesia, or dyskinesia. Atypical TTS types are also defined according to the localization of wall motion abnormalities and include the midventricular, basal and focal wall motion patterns (8).

Despite being an acute and reversible form of myocardial dysfunction, TTS is often associated with serious adverse in-hospital complications, namely acute heart failure, cardiogenic shock and life-threatening arrythmias. Given the absence of randomized clinical trials, management is based on empirical supportive pharmacological therapy, including HF treatment (9).

The exact pathophysiology of TTS is not fully understood, but there is evidence that intense sympathetic nervous system activation and high levels of catecholamines play a key role, as most patients have clinical signs of intense sympathetic activation. In this response, there are two elements involved. The first is the cognitive centers of the brain and hypothalamic-pituitary-adrenal axis, the perception of stress and the quantity of catecholamines released in response to each stress (i.e., the gain of the hypothalamic-pituitary-adrenal axis). The second is the response of the cardiovascular system (namely the myocardium, coronary arteries and peripheral vasculature) to the catecholamines surge (3).

TTS is usually triggered by emotional or physical stressors. Psychological triggers include a variety of emotions, including grief, interpersonal conflicts, fear and panic, anxiety, financial or employment problems, embarrassment and positive emotional events, such as a surprise party. Patients with TTS have an elevated prevalence of psychiatric disorders, namely depression, anxiety and type-D-personality, which is characterized by negative emotions and social inhibition. In particular, depressed patients show an exaggerated norepinephrine response to emotional stress, and some of them have an increased spillover and decreased reuptake of norepinephrine. Patients with panic disorders and anxiety also have a decreased catecholamine reuptake. Furthermore, antidepressants, namely selective norepinephrine reuptake inhibitors also increase local catecholamines levels. Consequently, patients with psychiatric disorders are more susceptible to TTS due to an increased sympathetic response to acute stress combined with greater cardiac sympathetic sensitivity. Moreover, TTS typically affects postmenopausal females and the sex disparity could be the consequence of different coping strategies for stress (8).

During the COVID-19 pandemic, an increased incidence of TTS in both the general population and COVID-19 patients has been reported. Before the pandemic, between 1.4 and 1.8% of patients admitted with suspected acute coronary syndrome were diagnosed with TTS, while during the COVID-19 pandemic this percentage increased to 7.75%. Proposed mechanisms for this association include generalized increases in psychological distress, the cytokine storm, increased sympathetic responses in COVID-19 patients, and microvascular dysfunction (10). In our case, a marked and intense emotional stress, due to extra work to replace quarantined colleagues, worked as a TTS trigger.

Other factors involved in the pathophysiology of TTS include estrogen deprivation, inflammation and immune activation (3). In line with the latter hypothesis, there is a potential link between rheumatological disorders and TTS (11). A retrospective epidemiologic study showed that the prevalence of TA among all TTS-related hospitalizations was 0.05%. Patients with both conditions were younger, predominantly Caucasian, had a higher prevalence of peripheral vascular disease, chronic obstructive pulmonary disease, psychiatric disorders, and smoking. During admission, patients with TA and TTS had a higher rate of stroke, respiratory failure, necessity of mechanical ventilation and higher healthcare resource utilization, highlighting the possible role of TA in the prognosis of TTS (12). However, given the paucity of reports linking TA and other autoimmune diseases with TTS even though both diseases have a predilection for middle-aged women, it has been suggested that immunosuppressive treatment of these rheumatological disorders might protect against pro-inflammatory responses to catecholamine surge seen in TTS (11).

The sympathetic stimulation and subsequent catecholamine surge involved in TTS can cause acute myocardial dysfunction through a variety of mechanisms, including microvascular dysfunction, multi-vessel epicardial coronary artery spasm, direct myocyte toxicity and activation of cardiomyocyte preservation pathways by downregulating cellular metabolism (11).

Coronary microvascular dysfunction might result from microvascular coronary vasoconstriction and myocardial stunning induced by sympathetic over-reactivity. While microvascular dysfunction is reversible in most patients with TTS, it has prognostic implications, being associated with poor long-term clinical outcome (13).

As in our case, epicardial coronary vasospasm has been reported during angiography in several patients with TTS and emotional stress reduces endothelium-dependent dilation. However, vasospasm could be an epiphenomenon following systemic exposure to high catecholamine levels. Furthermore, vasospasm would have to occur in the mid and distal segments of all major coronary arteries simultaneously to cause typical TTS, and it should also selectively involve side branches of major epicardial vessels to explain the basal and midventricular variants. In addition, in TTS the degree of troponin elevation is relatively modest and disproportionately low compared with the territory of dysfunctional myocardium, suggesting that mechanisms other than ischemic myocardial necrosis are involved. Finally, most patients with TTS do not show any evidence of epicardial spasm even with provocative tests. In conclusion, epicardial coronary vasospasm could contribute to the pathophysiology in a subset but not all patients with TTS (3, 8). However, given that these two entities have similar predisposing factors, it might be useful to perform a provocative test to rule out the presence of epicardial spasm in patients with TTS, as this might have therapeutic implications. While beta-blockers may provide some protection against recurrence of TTS due to attenuation of the catecholamine surge, they may also increase tendency to vasospasm, probably due to the blockade of coronary beta2-receptors, leaving alfa-adrenergic receptors unopposed. Therefore, if epicardial coronary vasospasm is induced, therapy with beta-blocker should be considered more carefully, and calcium-channel blocker therapy should be preferred (14).

On the other side, an alternative explanation for the coronary vasospasm is the myocardial bridging. In one report, myocardial bridging was an independent predictor of positive acetylcholine test and MINOCA, and was also associated with a higher rate of hospitalization attributable to angina (15). Some characteristics of the myocardial bridging such as the bridged length and percent systolic compression of myocardial bridging are predictors of provoked vasospasm (16).

Finally, there might also be a link between TA and coronary vasospasm. In one report, a 16-year-old boy with TA suffered a reverted cardiac arrest due to left main vasospasm (17). In another case report, 52-year-old female with TA had signs of coronary vasospasm while undergoing a rubidium PET myocardial perfusion scan with adenosine for investigation of angina. The authors postulate that high levels of endothelin-1 that are present in TA might have reduced adenosine-induced coronary vasodilation, contributing to vasospasm (18).

In conclusion, we report the first case showing coexistence of TA, TTS, AR and coronary vasospasm leading to acute HF and myocardial ischemia. As this is a case report and the pathophysiology behind each of these entities is not yet fully understood, we cannot assume causality. However, we highlight possible pathways and review published evidence supporting their relationship, but, at the same time, we underscore conceivable explanations for this unusual finding. In our opinion, inflammatory alterations secondary to TA, diastolic hypoperfusion due to severe AR and neuro-hormonal factors related to GAD might render myocardium and coronary arteries more reactive and susceptible to acute stressor, especially in an emotionally intense period, such as a pandemic. Nevertheless, this is an observational report, and further insights of pathophysiology, inflammatory and neuro-hormonal pathways involved in these conditions are needed.

## Data Availability Statement

The original contributions presented in the study are included in the article/Supplementary Material, further inquiries can be directed to the corresponding author.

## Ethics Statement

Ethical review and approval was not required for the study on human participants in accordance with the local legislation and institutional requirements. The patients/participants provided their written informed consent to participate in this study. Written informed consent was obtained from the patient for the publication of any potentially identifiable images or data included in this article.

## Author Contributions

IP and MM: patient follow-up during admission and writing the manuscript. NA: patient follow-up during admission and manuscript revision. CF: coronary angiography. AB and EC: cardiovascular imaging (cardiac magnetic resonance and echocardiography). ES, IM, and PA: manuscript revision. All authors contributed to the article and approved the submitted version.

## Conflict of Interest

The authors declare that the research was conducted in the absence of any commercial or financial relationships that could be construed as a potential conflict of interest.

## Publisher's Note

All claims expressed in this article are solely those of the authors and do not necessarily represent those of their affiliated organizations, or those of the publisher, the editors and the reviewers. Any product that may be evaluated in this article, or claim that may be made by its manufacturer, is not guaranteed or endorsed by the publisher.

## Supplementary Material

Video S1 Parasternal long axis view showing left ventricle dilation and dilation of aortic root and ascending aorta.

Video S2 Apical 4-chamber view revealing left ventricle dilation with reduced ejection fraction due to apical and midventricular akinesia in the septal and lateral walls, and normal right ventricular function.

Video S3 Severe aortic regurgitation in apical 5-chamber view.

Video S4 Apical 2-chamber view showing akinesia of the midventricular and apical segments of the anterior and inferior left ventricular walls.
